# Deep Probabilistic Learning Model for Prediction of Ionic Liquids Toxicity

**DOI:** 10.3390/ijms23095258

**Published:** 2022-05-09

**Authors:** Mapopa Chipofya, Hilal Tayara, Kil To Chong

**Affiliations:** 1 Department of Electronics and Information Engineering, Jeonbuk National University, Jeonju 54896, Korea; mapopachipofya@jbnu.ac.kr; 2 School of International Engineering and Science, Jeonbuk National University, Jeonju 54896, Korea; 3 Advanced Electronics and Information Research Center, Jeonbuk National University, Jeonju 54896, Korea

**Keywords:** small molecules, ionic liquids, toxicity, probabilistic deep learning, artificial intelligence

## Abstract

Identification of ionic liquids with low toxicity is paramount for applications in various domains. Traditional approaches used for determining the toxicity of ionic liquids are often expensive, and can be labor intensive and time consuming. In order to mitigate these limitations, researchers have resorted to using computational models. This work presents a probabilistic model built from deep kernel learning with the aim of predicting the toxicity of ionic liquids in the leukemia rat cell line (IPC-81). Only open source tools, namely, RDKit and Mol2vec, are required to generate predictors for this model; as such, its predictions are solely based on chemical structure of the ionic liquids and no manual extraction of features is needed. The model recorded an RMSE of 0.228 and $R^{2}$ of 0.943. These results indicate that the model is both reliable and accurate. Furthermore, this model provides an accompanying uncertainty level for every prediction it makes. This is important because discrepancies in experimental measurements that generated the dataset used herein are inevitable, and ought to be modeled. A user-friendly web server was developed as well, enabling researchers and practitioners ti make predictions using this model.

## 1. Introduction

Materials which exist in liquid phase at temperatures below 100 ${}^{\circ }C$ and are composed of organic or inorganic cations and anions are referred to as room temperature ionic liquids. Often, they are more loosely called ionic liquids (ILs). These materials exhibit a unique set of desirable properties, such as a low melting point, negligible volatility, thermal and chemical stability, high ionic conductivity, solubility with many compounds, low flammability, moderate viscosity, high polarity, and high recyclability [1,2,3,4]. Hence, they have drawn great interest as a research topic and found applications in various fields such as catalysis [5,6], pharmaceuticals [7,8], biopolymer processing [9], nuclear fuel reprocessing [10,11], solar thermal energy [12], and batteries [2,13]. However, there is a concern that, owing to their solubility in aqueous media, ionic liquids may interact with biota, distress it, and ultimately impact human health when these chemicals are discharged into the environment through wastewater [14].

Prominent research results regarding the toxic effects induced by ILs in the ecosystem are presented in the works of Samorı et al. [15] and Latała et al. [16]. Overall, studies leading to identification of more ILs with known effects on the environment have increased at a slower pace than anticipated [14]. The usual and most effective way of conducting experiments to measure the toxicity of ILs directly with the aim of determining ILs with desirable low toxicity has been deemed time-consuming, resource-intensive, and even impractical due to the large number of feasible combinations between cations and anions [14,17]. To quickly build on the available results obtained from experimental measurements and mitigate the limitations associated with conducting further experimental measurements, computational methods, which often involve machine learning, have become a preferred tool. Herein, we consider several computational tools that have been developed recently to predict the toxicity of ILs against the leukemia rat cell line IPC-81. IPC-81 has been frequently used to quantitatively indicate the toxicity of ILs [14,18,19,20,21,22,23,24,25].

Wang et al. [17] developed a support vector machine (SVM) model based on a dataset containing 355 ILs. From their respective simplified molecular-input line entry system (SMILES) strings, nine cation descriptors, nine anion descriptors, and 24 general descriptors were obtained for each IL using a feature extraction algorithm [26] and the RDKit cheminformatics tool [27]. Their feature extraction algorithm uses a predefined set of substructures which act as descriptors. The frequency with which each descriptor appears in the IL molecule is then used as input to the model, similar to group contribution (GC)-based methods [28,29,30,31,32]. The SVM model trained in this way yielded a satisfactory RMSE of 0.2875 on the 355 ILs.

More recently, Kang et al. [33] embarked on improving traditional GC-based approaches to predicting the toxicity of ionic liquids [34,35]. They developed a novel method, termed atom surface fragment contribution (ASFC), which uses the surface area of screening charge density ($S_{\sigma \mathrm{-surface}}$) calculated based on quantum chemistry. Unlike in GC, where only the types and frequencies of functional groups are considered and interactions between groups are ignored (thus rendering isomeric groups indistinguishable [34]), ASFC has the capability to distinguish the contributions of each group in different molecules, and hence the potential to improve the reliability of GC models [33]. In ASFC, the $S_{\sigma \mathrm{-surface}}$ values of atoms are obtained using BIOVIA COSMOtherm 2020 software, which contains COSMO files of 74 cations and 15 anions from the quantum chemical level of BP-TZVPD-FINE. The $S_{\sigma \mathrm{-surface}}$ values for groups were found by summing the $S_{\sigma \mathrm{-surface}}$ of all atoms in each group. Group $S_{\sigma \mathrm{-surface}}$ values were used as predictor descriptors in a multiple linear regression (MLR) model similar to the one used by Hossain et al. [36]. The $R^{2}$ and MSE of the ASFC model were 0.924 and 0.071, respectively.

The models described above have shown an exceptional ability to predict toxicity with great accuracy and reliability by taking into account expert information regarding the creation of predictor descriptors. However, it may be difficult for someone who has no or little domain expertise to create such specialized descriptors in order to use them when making predictions concerning new ILs. Second, several of the models described above employed commercial software such as COSMOtherm to extract the desired features, which adds to their cost. Lastly, all these models are deterministic; they do not model uncertainty in either the data nor in the models themselves. Kang et al. [33] noted that there might be experimental errors in the set of ionic liquids that they used in their work. It is therefore crucial that the uncertainty associated with the data be included in the model.

Consequently, this work aims to achieve three main goals. First, we intend to use existing open-source software to generate descriptors for predicting toxicity of ionic liquids towards the leukemia rat cell line in a way that requires no or very little domain expertise. Second, based on these features, we intend to build an accurate and reliable probabilistic deep learning model for predicting toxicity. Such a model should be capable of capturing aleatoric uncertainty, which is the uncertainty due to irreducible noise in the data. Aleatoric uncertainty models the stochastic nature of the process of generating data [37]. Lastly, we built a web tool for the ensuing model to allow other researchers and practitioners to use it in their work.

## 2. Materials and Methods

### 2.1. Data Preparation

**Figure 1:**
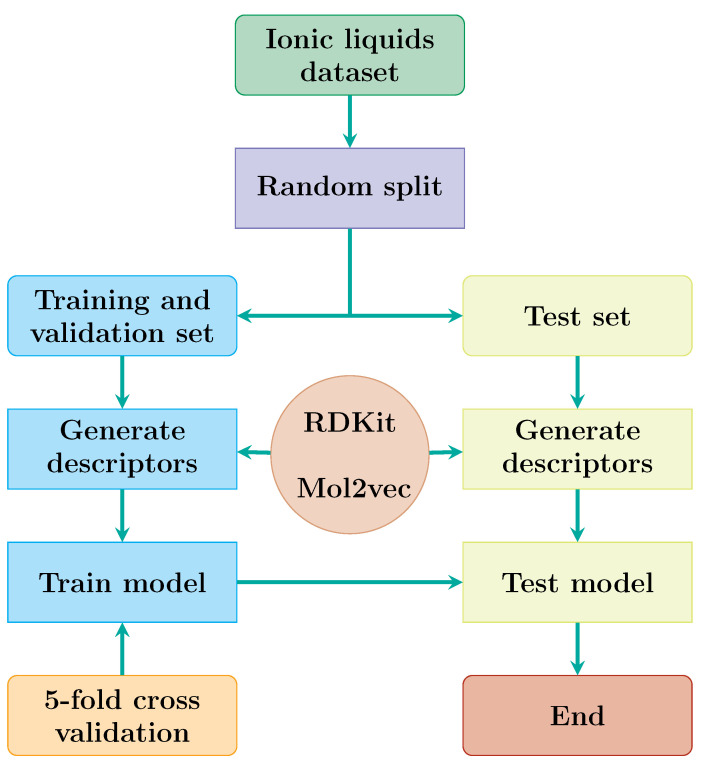
Workflow showing how the data were split, features generated, and cross validation used in modeling toxicity prediction for ionic liquids.

A dataset containing 155 ionic liquids which exhibit toxicity towards the leukemia rat cell line IPC-81 was collected from the literature [33,38]. The logarithm of half maximal effective concentration, $\log{\mathrm{EC}}_{50}$, was used to represent the toxicity level, whereas the SMILES string for each ionic liquid was used to generate the features used for modeling. The dataset was split randomly into subsets, which contained 140 ionic liquids for training and cross-validation and 15 for testing. Figure 1 depicts the overall process.

### 2.2. Molecular Descriptors and Features

**Figure 2:**
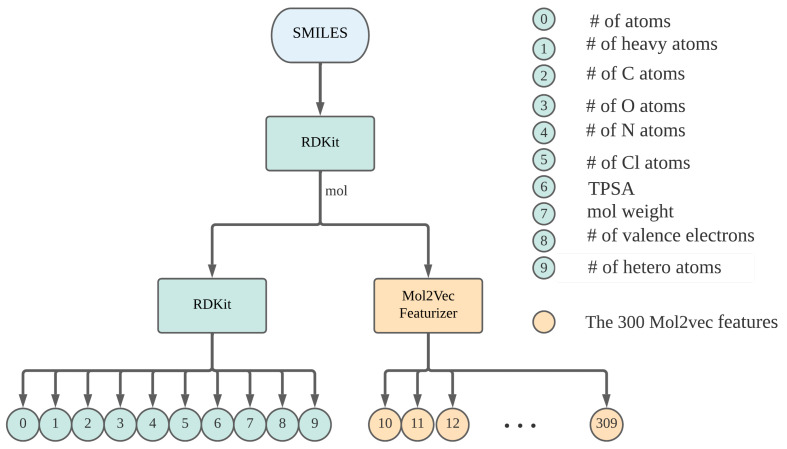
Workflow showing how ten features were generated from RDKit and 300 from Mol2vec for each of the ionic liquids based on their respective SMILES strings.

A total of 310 features were used to describe the physical and chemical properties of each of the ionic liquids. In particular, the first ten features were obtained from RDKit molecular descriptors. These descriptors were the number of atoms in the molecule, number of heavy atoms, number of carbon atoms, number of oxygen atoms, number of nitrogen atoms, number of chlorine atoms, the topological polar surface area (TPSA) of the molecule, the molecular weight, the number of valence electrons, and the number of heteroatoms for a molecule. The rest of the features (300) were obtained using a pretrained Mol2vec [39] model. Mol2vec is an unsupervised machine learning approach to learning the vector representations of molecular substructures. The pretrained Mol2vec model used in this experiment was reported to have been trained in an unsupervised fashion on 19.9 million compounds from the ZINC version 15 [40,41] and chEMBL version 23 [42] databases. The ten features from RDkit and the 300 features from Mol2vec were then concatenated to produce one feature vector with a length of 310. Figure 2 depicts the workflow for generating these 310 features for each ionic liquid.

### 2.3. Deep Kernel Learning

**Figure 3:**
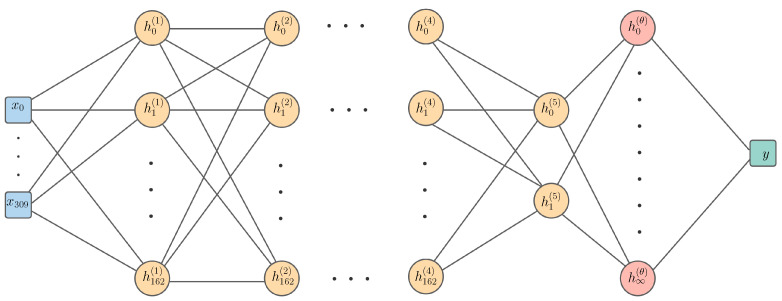
A Gaussian process with a deep kernel which maps the 310 input features x through five parametric hidden layers followed by a single hidden layer with an infinite number of basis functions using the RBF base kernel. The kernel’s hyperparameters are denoted as θ, whereas those of the parametric layers are denoted as w. Each of the first four parametric hidden layers has 163 units, while the final parametric hidden layer has two units. There is only one unit in the output y, representing a single value for $\log{\mathrm{EC}}_{50}$.

A deep kernel model can be thought of as applying a Gaussian process with a base kernel kθ to the final hidden layer of the deep neural network. In effect, this means that the deep neural network has a hidden layer with an infinite number of hidden units, as a Gaussian process with a base kernel kθ, such as the radial basis function (RBF) kernel, corresponds to an infinite basis function representation [43]. Figure 3 shows the pedagogical architecture of the deep kernel learning model used in our experiments.

From an RBF base kernel $k(x_{i},x_{j}|\theta )$ with parameters θ, the input features x are transformed, using a probabilistic Gaussian process, as

(1) $$k\left(x_{i},x_{j}|\theta \right)\to k\left(g\left(x_{i},w\right),g\left(x_{j},w\right)|\theta ,w\right)$$

where g(x,w) is the nonlinear mapping provided by the deep neural network. The hyperparameters of the deep neural network, w, and those of the base kernel, θ, are combined as $\gamma =\left\{w,\theta \right\}$ and learnt jointly by maximizing the log marginal likelihood L of the targets y, as follows:

(2) $$\log p\left(y|\gamma ,X\right)\propto -\left[y^{⊤}{\left(K_{\gamma }+\sigma^{2}I\right)}^{-1}y+\log\left|K_{\gamma }+\sigma^{2}I\right|\right].$$

To learn the kernel, the chain rule is applied to compute

(3) $$\frac{\partial L}{\partial \theta }=\frac{\partial L}{\partial K_{\gamma }}\frac{\partial K_{\gamma }}{\partial \theta }$$

(4) $$\frac{\partial L}{\partial w}=\frac{\partial L}{\partial K_{\gamma }}\frac{\partial K_{\gamma }}{\partial g(x,w)}\frac{\partial g(x,w)}{\partial w}$$

where the implicit derivative of the log marginal likelihood with respect to the data covariance matrix $K_{\gamma }$ is provided by

$$\frac{\partial L}{\partial K_{\gamma }}=\frac{1}{2}\left(K_{\gamma }^{-1}{\mathrm{yy}}^{⊤}K_{\gamma }^{-1}-K_{\gamma }^{-1}\right).$$

For scalability, a structured kernel interpolation [44] covariance matrix, $K_{\mathrm{SKI}}$, is used instead of $K_{\gamma }$:

(5) $$K_{\gamma }\approx WK_{U,U}W^{⊤}:=K_{\mathrm{SKI}}$$

where *U* is the set of grid inducing points, $K_{U,U}$ is the kernel matrix between the inducing points, and *W* is a sparse matrix of the interpolation weights.

### 2.4. Training Details and Model Hyperparameters

We used the GPyTorch [45] library to implement the model described in the deep kernel learning (DKL) section. To obtain optimal model hyperparameters, we used the Optuna hyperparameter optimization framework [46]. Table 1 contains more information about the model’s implementation and its associated hyperparameters.

With the hyperparameters fixed as shown in the optimal setting column of Table 1, a DKL model was developed using the training set and a five-fold cross-validation scheme, as depicted in Figure 1. A representative model was selected based on the optimal performance during cross-validation. Table S1 in the Supplementary Materials shows the results of cross-validation and which instance of the model was selected. The selected model was then evaluated on the test dataset.

### 2.5. Performance Evaluation Metrics

To evaluate the performance of the model, we used standard statistical metrics that are commonly used on regression problems. These metrics were the mean squared error (MSE), root mean squared error (RMSE), coefficient of determination($R^{2}$), and average absolute relative deviation (AARD).

With *N* samples of data where the measured $\log{\mathrm{EC}}_{50}$ from experiments for sample *i* is provided by $y_{i}^{exp}$ and the corresponding prediction from the DKL model by $y_{i}^{pred}$, the aforementioned metrics can be obtained as follows:

(6) $$\mathrm{MSE}=\frac{1}{N}\sum_{i=1}^{N}{\left(y_{i}^{pred}-y_{i}^{exp}\right)}^{2}$$

(7) $$\mathrm{RMSE}=\sqrt{\frac{1}{N}\sum_{i=1}^{N}{\left(y_{i}^{pred}-y_{i}^{exp}\right)}^{2}}$$

(8) $$R^{2}=1-\frac{\sum_{i=1}^{N}{\left(y_{i}^{pred}-y_{i}^{exp}\right)}^{2}}{\sum_{i=1}^{N}{\left(y_{i}^{pred}-\bar{y}\right)}^{2}}$$

(9) $$\mathrm{AARD}=\frac{1}{N}\sum_{i=1}^{N}\left|\frac{y_{i}^{pred}-y_{i}^{exp}}{y_{i}^{exp}}\right|$$

Note that the term $\bar{y}$ in Equation (8) represents the average measured $\log{\mathrm{EC}}_{50}$ in the dataset.

## 3. Results and Discussion

In this section, we provide results showing the performance of the DKL model and compare it with GC and ASFC models, two of the state-of-the-art models in this area. These two models, especially ASFC, have been shown to be accurate and reliable in predicting the toxicity of ionic liquids towards the leukemia rat cell line IPC-81. Here, we determine whether DKL can be as accurate and reliable as ASFC.

Table 2 compares the performance of the DKL model with the existing models GC and ASFC. On the 140 ionic liquids used for cross-validation, DKL performs well in all metrics compared to both GC and ASFC. In particular, DKL achieves an RMSE of 0.233, which is about 10% lower than the RMSE achieved by ASFC. The determination coefficient, $R^{2}$, achieved by DKL was 0.94, compared to 0.93 for ASFC and 0.924 for GC.

Similarly, on the full dataset containing 155 ionic liquids DKL achieved an RMSE of 0.228, compared to 0.294 achieved by ASFC, representing an improvement of about 22%. The coefficient of determination rose from 0.911 for ASFC to 0.943 for DKL.

It is important to note that on both sets of results DKL achieved an RMSE of around 0.23 and an $R^{2}$ of about 0.94. This is in contrast to deviations of 0.256–0.294 in RMSE and 0.93–0.911 in $R^{2}$ achieved by ASFC, which are slightly larger. The minor deviations in the scores obtained by DKL could mean that the model was not overfitted, and is thus better able to generalize.

**Figure 4:**
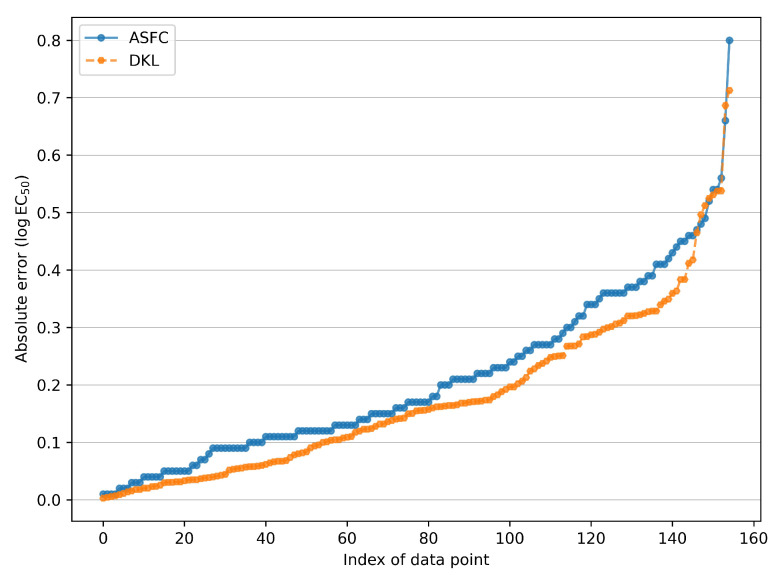
Sorted absolute errors between experimental and predicted $\log{\mathrm{EC}}_{50}$ for ASFC and DKL models on the 155 ionic liquids which form the entire dataset.

The contrast in the performance of ASFC and DKL can further be discerned in Figure 4. The figure shows the sorted absolute errors between experimental and predicted $\log{\mathrm{EC}}_{50}$ for ASFC and DKL models on the full dataset of 155 ionic liquids. The area under the absolute error curve associated with DKL is evidently smaller than that of ASFC, revealing a higher predictive accuracy for IL toxicity with the DKL model.

**Figure 5:**
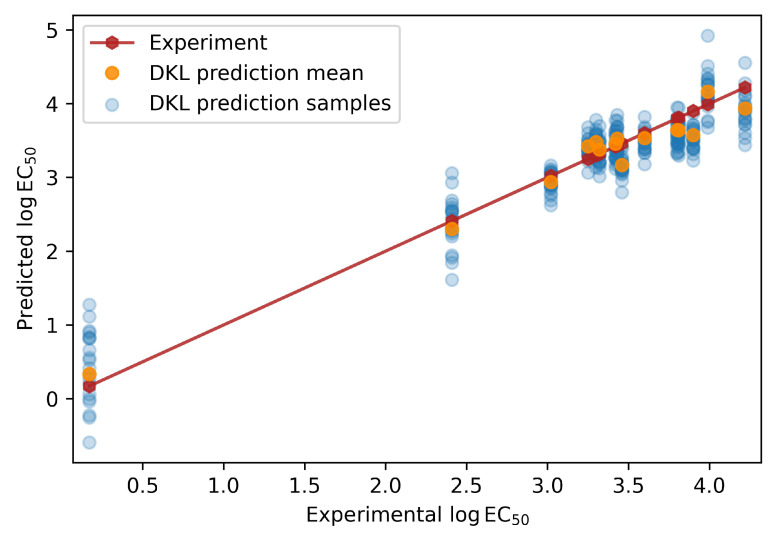
Comparison between experimental and DKL predicted $\log{\mathrm{EC}}_{50}$ for the fifteen ionic liquids in the test dataset.

Because the DKL model is a probabilistic model, it can be used to make predictions for any number of samples while observing the mean predictions and covariances. This information can then be used to determine the uncertainty in the predictions made by the model. Figure 5 shows a comparison between experimental and predicted $\log{\mathrm{EC}}_{50}$ for the fifteen ionic liquids that formed the test dataset. It can be observed that the mean predictions made by DKL are close to the experimental $\log{\mathrm{EC}}_{50}$ values. This demonstrates that the model learned well and can make authentic predictions. More importantly, we can query the model to show a number of samples that contribute to this prediction, from which we can visually determine the levels of uncertainty in the model. In Figure 5, we show twenty such samples for each of the fifteen predictions.

**Figure 6:**
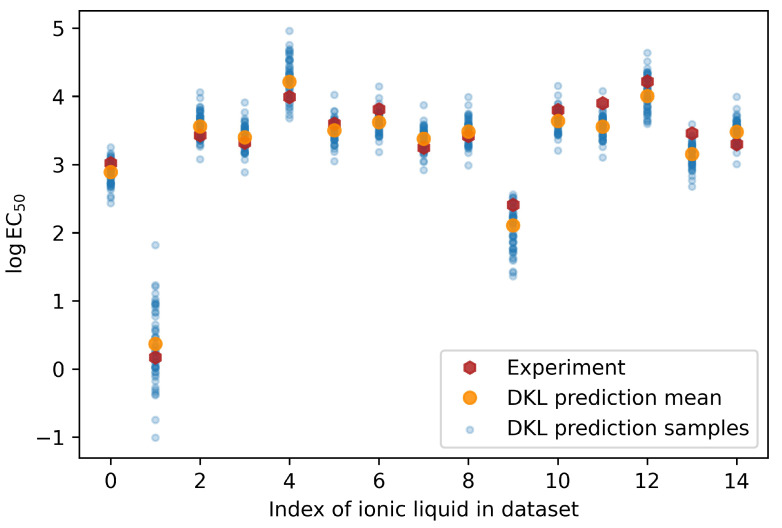
Comparisons between experiment and DKL predicted $\log{\mathrm{EC}}_{50}$ for each of the fifteen ionic liquids forming the test dataset. For each DKL prediction, we drew samples that contribute to the mean prediction.

Figure 6 shows the $\log{\mathrm{EC}}_{50}$ values predicted by the DKL model in comparison with the values measured by experiment for the same fifteen ionic liquids used in the test dataset, this time using the indices of the ILs in the dataset as the x-axis variable. From the figure, it can be observed that the model is more uncertain for ILs at index 1, while being more certain about other predictions, such as the prediction at index 0. Such information is important in allowing practitioners or researchers to make decisions about the predictions made by the model. Consider a situation where the chemical structure of the ionic liquid being evaluated is very similar to two other ILs which have very different levels of toxicity, and the latter two were used for modeling. Ideally, the model’s uncertainty should be high in order to reflect the varied toxicity levels of the data on which it was modeled. If the uncertainty range enters regions where the toxicity levels are unacceptable, the practitioner may conduct further experiments or gather more information from other sources in order to obtain additional insight about the IL. This extra information would then lead to deciding whether or not to proceed with use of the IL in the intended application.

### 3.1. Applicability Domain

As per Organisation for Economic Co-operation and Development (OECD) principles which stipulate that Quantitative Structure–Activity Relationship (QSAR) prediction models should have well-defined applicability domains (AD), we performed an AD analysis for this study. We used the standardization technique (ST) proposed by Roy et al. [47]. In an ideal situation, data are distributed such that 99.7% of the population falls within the range mean ± 3 standard deviations (SD). In this context, this implies that mean ± 3SD represents the zone to which the majority of the ionic liquids in the training set belong. Any ionic liquids appearing outside this region are considered to be different from the rest of the ionic liquids.

In ST, a descriptor column is standardized based on the corresponding mean and standard deviation for the training set only. If the ensuing standardized value for a descriptor of a particular ionic liquid is more than 3.0, then the ionic liquid is considered an outlier if it is in the training set, and is considered outside the AD if it is part of the test set [48]. The applicability domain section in the Supplementary Material provides a full description of the ST algorithm.

**Figure 7:**
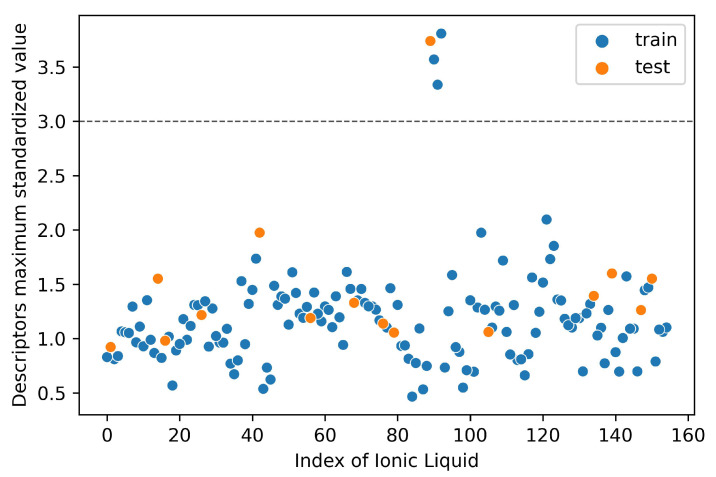
Applicability domain defined in this study. One ionic liquid in the test set is outside the AD, and three ILs in the training set are outliers as their corresponding descriptors’ maximum standardized values are greater than the 3.0 threshold.

The distribution map of the applicability domain is shown in Figure 7. The coverage of the test set in the applicability domain using the ST shows that all but one ionic liquid fell outside the AD. Similarly, in the training set, three of the 140 ionic liquids were considered outliers. This means that 93% and 98% of the ionic liquids in the test and training sets, respectively, fall within the AD.

Our DKL model uses the “mixtures out” validation protocol. To a large extent, this protocol estimates the ability of models to predict new combinations of anions and cations. This may provide overoptimistic results, as described elsewhere [49,50]. This applies to the ASFC model with which we are comparing DKL in this study as well. There exist more rigorous validation protocols, such as “components validation”, which can test the model’s prediction of new types of ions. By design, components validation is more similar to real-life situations [49,51]. Thus, replacing mixtures validation with components validation in our workflow may minimize the level of optimistic results, if any.

### 3.2. Prediction Web Server

A web server that encapsulates the DKL model was built. The tool accepts SMILES strings as input for the ionic liquids and provides results in both tabular and interactive visualization formats. The server is publicly available at http://nsclbio.jbnu.ac.kr/tools/iltox/, accessed on 8 April 2022.

## 4. Conclusions

Currently available data do not show that ionic liquids are environmentally safe chemicals; as such, their toxicity risk has to be evaluated in order to ensure their safe use in a wide range of applications. In this work, we have presented a probabilistic deep learning model that can be used to predict the toxicity of ionic liquids towards the leukemia rat cell-line (IPC-81) reliably and accurately. The model pipeline requires little or no expert domain knowledge in the generation of features to be used for subsequent predictions. In addition, all predictors are generated using open source cheminformatics tools. In addition, because the model is embedded with a Gaussian process it has the inherent capability to attach a level of uncertainty to each prediction it makes. As the dataset used in this work was generated from experimental measurements in which inconsistencies are, at the very least, unavoidable, the uncertainty associated with these data had to be addressed. In that respect, the results obtained here indicate that the presented probabilistic deep learning model represents a good choice. Furthermore, the probabilistic nature of the model means that it provides vital information with which users can interpret prediction results and gain insight about both the data and the model. Finally, based on this model we developed a web-based tool which can be used to make predictions. This tool is freely available on our project website.

## Figures and Tables

**Table 1:** Hyperparameters for the deep kernel model used in our experiments.

Hyperparameter	Options	Optimal Setting
Basis kernel function	RBF	RBF
Grid size	16 to 100	35
Learning rate	1 × 10 ^−5^ to 3 × 10 ^−1^	0.0130925
Optimizer	SGD or RMSprop or Adam	RMSprop
Deep neural network (DNN) layers	2 to 7	5
Units in each layer (except the last)	32 to 512	163
Units in the last layer of the DNN	2	2
Activation function	ReLU or LeakyReLU or Tanh	LeakyReLU

**Table 2:** Performance comparison of our DKL model with existing models on the training, validation, and full datasets.

Model	Dataset	Samples	AARD%↓	$R^{2}$↑	MSE↓	RMSE↓
GC	train + valid	140	11.358	0.924	0.071	0.267
ASFC	train + valid	140	10.898	0.930	0.065	0.256
DKL	train + valid	140	8.756	0.940	0.054	0.233
GC	full	155	-	-	-	-
ASFC	full	155	10.613	0.911	0.086	0.294
DKL	full	155	8.932	0.943	0.052	0.228

## Data Availability

The dataset used in this work can be found in the attached Supplementary Material and can be downloaded from the project web page at http://nsclbio.jbnu.ac.kr/tools/iltox/, accessed on 8 April 2022.

## Supplementary Materials

The following are available online at https://www.mdpi.com/article/10.3390/ijms23095258/s1.

## Abbreviations

The following abbreviations are used in this manuscript:

EC	Effective Concentration
GC	Group Contribution
DKL	Deep Kernel Learning
MSE	Mean Squared Error
SVM	Support Vector Machine
AARD	Average Absolute Relative Deviation
ASFC	Atom Surface Fragment Contribution
OECD	Organisation for Economic Co-operation and Development
RMSE	Root Mean Squared Error
QSAR	Quantitative Structure–Activity Relationship
SMILES	Simplified Molecular-Input Line Entry System
