# Supplementary material for: Deep Probabilistic Learning Model for Prediction of Ionic Liquids Toxicity
Source: Int J Mol Sci. 2022 May 9;23(9):5258. doi: 10.3390/ijms23095258 (PMC9104997; doi:10.3390/ijms23095258)
Supplement: Supplementary file 1 [file ijms-23-05258-s001.zip › ijms-1688912-supplementary.pdf]

Supplementary Material

Deep probabilistic learning model for prediction of ionic liquids toxicity

Mapopa Chipofya, Hilal Tayara and Kil To Chong

Cross-validation results

Fold	AARD% ↓	$\mathbf{R^2}$↑	MSE↓	RMSE↓
1	7.8829	0.9601	0.0363	0.1906
2	6.2297	0.9544	0.0415	0.2036
3	12.0389	0.9054	0.0861	0.2934
4	9.1174	0.9400	0.0546	0.2337
5	6.7110	0.9534	0.0424	0.2059
Average	8.3960 ± 2.0778	0.9426 ± 0.0198	0.0522 ±0.0180	0.2254 ± 0.0368

Table S1: Results of cross validation. The instance of the model from fold 4 was chosen as its results are closer to the overall average results considering all the four metrics.

Applicability Domain

A simple and user friendly standardization technique for applicability domain (AD) estimation was proposed in [1]. This approach can be used to detect outliers in the training set and identify compounds residing outside the AD in the test set. This helps to build reliable and acceptable QSAR models. This approach is also transparent and reproducible [1].

The standardization technique involves two main stages. The first stage is about standardizing the descriptors in the training and test sets using Algorithm 1 [1, 2]. Once all the descriptors are normalized, Algorithm 2 determines a representative standardized descriptor value for each ionic liquid. If the ensuing value is greather than 3.0, the corresponding ionic liquid is considered to be outside the applicability domain if it is in the test set or an outlier if it is part of the training set. On the other hand, a value less than or equal to 3.0 implies that the concerned ionic liquid is within the applicability domain if it is in the test set, and not considered an outlier if it belongs to the training set [1, 2].

Data: training and test set descriptors
Result: Standardized descriptors of ionic liquids

Total number of descriptors, N_D
Total number of ILs, N_L

for $k = 1, 2, 3,\ \dots\ N_L$ **do**
  for $i = 1, 2, 3,\ \dots\ N_D$ **do**
    Standardize all descriptors in the training and test set using

$$S_{ki} = \frac{|X_{ki} - \bar{X}_i|}{\sigma_{X_i}}$$

      X_{ki} is the original descriptor i for IL k
      $\bar{X}_i$ is the mean value of descriptor X_i for training set only
      σ_{X_i} is the standard deviation of descriptor X_i for training set only
  end
end

Algorithm S1: Standardizing descriptors.

Data: Standardized training and test descriptors
Result: A set of ionic liquids that are within the applicability domain
A set of ionic liquids that fall outside the applicability domain
A set of ionic liquids that are outliers

Total number of ionic liquids, N_L
Index i as defined in Algorithm 1

```
for k = 1, 2, 3, ... N_L do
    [S_i]_max(k) = maximum S_ki value for IL k ;
    if [S_i]_max(k) ≤ 3.0 then
        if the ionic liquid is in the training set then
            the ionic liquid is not an outlier;
        else
            the ionic liquid is within the applicability domain;
        end
    else
        [S_i]_min(k) = minimum S_ki value for ionic liquid k ;
        if [S_i]_min(k) > 3.0 then
            if the ionic liquid is in the training set then
                the ionic liquid is an outlier;
            else
                the ionic liquid is outside the applicability domain;
            end
        else
            S_new(k) = S̄_k + 1.28 × σ_s_k ;
                S̄_k is the mean of S_ki values for ionic liquid k
                σ_s_k is the standard deviation of S_ki values for
                ionic liquid k

            if S_new(k) ≤ 3.0 then
                if the ionic liquid is in the training set then
                    the ionic liquid is not an outlier;
                else
                    the ionic liquid is within applicability domain;
                end
            else
                if the ionic liquid is in the training set then
                    the ionic liquid is an outlier;
                else
                    the ionic liquid is outside the applicability domain;
                end
            end
        end
    end
end
```

Algorithm S2: Standardization technique for applicability domain.

Dataset

Ionic Liquid	Experiment	ASFC	DKL
1-Benzyl-3-methylimidazolium tetrafluoroborate	2.97	3.09	3.08
1-butyl-3,4-dimethylpyridinium tetrafluoroborate	3.02	2.91	2.94
1-butyl-3-methylpyridinium tetrafluoroborate	3.3	3.16	3.14
1-butylpyridinium tetrafluoroborate	3.16	3.21	3.2
1-butylquinolinium tetrafluoroborate	2.16	1.82	2.33
1-ethyl-3-methylimidazolium tetrafluoroborate	3.44	3.71	3.63
1-hexyl-4-methylpyridinium tetrafluoroborate	2.17	2.53	2.48
1-octyl-4-methylpyridinium tetrafluoroborate	1.49	1.9	1.59
3-hexyl-1,2-dimethylimidazolium tetrafluoroborate	1.9	2.22	2.1
3-methyl-1-octylimidazolium tetrafluoroborate	1.59	1.84	2.01
3-methyl-1-propylimidazolium tetrafluoroborate	3.45	3.42	3.49
1-decyl-3-methylimidazolium tetrafluoroborate	0.77	1.2	0.95
1-heptyl-3-methylimidazolium tetrafluoroborate	2.58	2.17	2.42
1-hexyl-3-methylimidazolium tetrafluoroborate	2.98	2.44	2.77
1-octylquinolinium tetrafluoroborate	0.17	0.47	0.33
1-butyl-1-methylpiperidinium bromide	4.03	3.95	3.9
1-butyl-3-methylimidazolium bromide	3.43	3.55	3.53
1-butylpyridinium bromide	3.9	3.69	3.58
1-butylquinolinium bromide	2.32	2.3	2.83
1-Pentylpyridinium bromide	3.15	3.37	3.31
1-butyl-1-methylpyrrolidinium bromide	3.77	3.91	3.83
1-Methyl-3-pentylimidazolium chloride	3.16	3.04	2.99
1-(3-methoxypropyl)-1-methylpiperidinium chloride	4.4	3.84	4.41
1-(Ethoxymethyl)-1-methylpiperidinium chloride	4.24	4.25	4.28
1-(ethoxymethyl)pyridinium chloride	3.32	3.78	3.86
1-butyl-3-methylimidazolium chloride	3.55	3.34	3.39
1-Butyl-4-methylpyridinium chloride	3.32	3.41	3.38
1-Butylpyridinium chloride	3.77	3.48	3.43
1-heptyl-3-methylimidazolium chloride	2.53	2.44	2.43
1-hexadecyl-3-methylimidazolium chloride	-0.24	-0.42	0.3
1-hexyl-1-methylpyrrolidinium chloride	2.93	3.02	3.1
1-Hexyl-3-methylpyridinium chloride	2.4	2.77	2.81
1-Hexyl-4-methylpyridinium chloride	2.67	2.8	2.87
1-Hexylpyridinium chloride	2.8	2.81	2.88
1-methyl-1-octylpyrrolidinium chloride	2.59	2.43	2.52
3-methyl-1-nonylimidazolium chloride	1.4	1.79	1.52
3-methyl-1-octylimidazolium chloride	2	2.11	2
benzyltetradecyldimethylammonium chloride	0.16	-0.12	0.3
4-(dimethylamino)-1-butylpyridinium chloride	1.94	2.06	1.96
1-butyl-1-methylpyrrolidinium dicyanamide	4.23	3.88	4.19
1-butyl-3-methylimidazolium dicyanamide	3.15	3.51	3.2
1-butyl-3-methylimidazolium hydrogensulfate	3.29	3.4	3.6
1-ethyl-3-methylimidazolium hydrogensulfate	3.99	4.03	4.16
1-(2-hydroxyethyl)-1-methylpiperidinium iodide	4.58	4.41	4.63

1-(2-hydroxyethyl)pyridinium iodide	4.16	4.26	4.16
1-butyl-3-methylimidazolium iodide	3.48	3.55	3.58
1-Ethyl-3-methylimidazolium methanesulfonate	3.97	3.93	3.82
1-Butylpyridinium methylsulfate	3.92	3.62	3.84
1-butyl-3-methylimidazolium 1-methylsulfate	3.21	3.48	3.53
1-butyl-3-methylimidazolium hexafluorophosphate	3.1	3.33	3.37
1-ethyl-3-methylimidazolium hexafluorophosphate	3.92	3.97	3.81
1-hexyl-3-methylimidazolium hexafluorophosphate	2.91	2.7	2.87
1-(2-ethoxyethyl)-1-methylpiperidinium bis(trifluoromethylsulfonyl)amide	3.34	3.22	3.4
1-(2-ethoxyethyl)pyridinium bis(trifluoromethylsulfonyl)amide	3.26	2.95	3.4
1-(2-hydroxyethyl)-1-methylpiperidinium bis(trifluoromethylsulfonyl)amide	3.65	3.77	3.61
1-(2-methoxyethyl)-1-methylpiperidinium bis(trifluoromethylsulfonyl)amide	3.25	3.63	3.48
1-(3-hydroxypropyl)-1-methylpyrrolidinium bis(trifluoromethylsulfonyl)amide	3.6	3.45	3.53
1-(3-methoxypropyl)-1-methylpiperidinium bis(trifluoromethylsulfonyl)amide	3.27	3.42	3.39
1-(3-methoxypropyl)pyridinium bis(trifluoromethylsulfonyl)amide	3.38	3.16	3.29
1-(cyanomethyl)-1-methylpiperidinium bis(trifluoromethylsulfonyl)amide	3.95	3.92	3.62
1-(ethoxymethyl)-1-methylpiperidinium bis(trifluoromethylsulfonyl)amide	3.41	3.82	3.53
1-butyl-1-methylpiperidinium bis(trifluoromethylsulfonyl)amide	3.41	3.32	3.25
1-butyl-3-methylimidazolium bis(trifluoromethylsulfonyl)amide	2.68	2.92	3
1-Hexyl-1-methylpyrrolidinium bis(trifluoromethylsulfonyl)amide	2.56	2.59	2.78
1-Pentylpyridinium bis(trifluoromethylsulfonyl)amide	2.85	2.74	2.84
1-Propylpyridinium bis(trifluoromethylsulfonyl)amide	3.2	3.37	3.37
3-methyl-1-octylimidazolium bis(trifluoromethylsulfonyl)amide	1.64	1.69	1.58
4-(2-ethoxyethyl)-4-methylmorpholinium bis(trifluoromethylsulfonyl)amide	3.69	3.22	3.57
4-(2-methoxyethyl)-4-methylmorpholinium bis(trifluoromethylsulfonyl)amide	3.81	3.72	3.64
4-(3-hydroxypropyl)-4-methylmorpholinium bis(trifluoromethylsulfonyl)amide	3.53	3.68	3.56
4-(3-methoxypropyl)-4-methylmorpholinium bis(trifluoromethylsulfonyl)amide	3.77	3.57	3.5
4-(ethoxymethyl)-4-methylmorpholinium bis(trifluoromethylsulfonyl)amide	3.36	3.81	3.61
4-butyl-4-methylmorpholinium bis(trifluoromethylsulfonyl)amide	3.43	3.41	3.39
1-(2-ethoxyethyl)-1-methylpyrrolidinium bis(trifluoromethylsulfonyl)amide	3.2	3.15	3.55
1-(2-hydroxyethyl)-3-methylimidazolium bis(trifluoromethylsulfonyl)amide	3.76	3.55	3.29
1-(2-methoxyethyl)-1-methylpyrrolidinium bis(trifluoromethylsulfonyl)amide	3.3	3.5	3.62
1-(2-methoxyethyl)-3-methylimidazolium bis(trifluoromethylsulfonyl)amide	3.25	3.35	3.42
1-butyl-1-methylpyrrolidinium bis(trifluoromethylsulfonyl)amide	3.01	3.28	2.87
4-(dimethylamino)-1-butylpyridinium bis(trifluoromethylsulfonyl)amide	1.75	1.64	1.69
1-butyl-3-methylimidazolium thiocyanate	3.42	3.51	3.46
1-ethyl-3-methylimidazolium thiocyanate	4.23	4.14	3.99
4-ethyl-4-methylmorpholinium toluene-4-sulfonate	3.81	3.98	3.73
1-ethyl-3-methylimidazolium toluene-4-sulfonate	3.81	3.56	3.57
1-ethyl-3-methylimidazolium trifluoroacetate	4	4.12	3.81
1-butyl-3-ethylimidazolium trifluoroacetate	3.31	3.19	3.32
1-Butylpyridinium trifluoromethanesulfonate	3.66	3.49	3.3
1-ethyl-3-methylimidazolium trifluoromethanesulfonate	4.09	3.99	3.74
1-Hexylpyridinium trifluoromethanesulfonate	2.54	2.82	2.65
1-butyl-3-ethylimidazolium trifluoromethanesulfonate	3.43	3.06	3.28
1-butyl-1-methylpyrrolidinium trifluorotris(pentafluoroethyl)phosphate	2.41	2.52	2.3
1-butyl-3-methylimidazolium trifluorotris(pentafluoroethyl)phosphate	1.81	2.15	2.1
1-ethyl-3-methylimidazolium trifluorotris(pentafluoroethyl)phosphate	3.23	2.79	2.52
1-hexyl-3-methylimidazolium trifluorotris(pentafluoroethyl)phosphate	1.53	1.52	1.44
1-butyl-3-ethylimidazolium tetrafluoroborate	3.26	2.78	3.29

1-methyl-3-pentylimidazolium tetrafluoroborate	3.09	2.77	3.36
1,1-dihexylpyrrolidinium tetrafluoroborate	1.23	1.21	1.29
1-butyl-3,5-dimethylpyridinium tetrafluoroborate	3.25	3.05	3.12
4-(dimethylamino)-1-ethylpyridinium bromide	2.9	2.73	2.88
1-decyl-3-methylimidazolium chloride	1.34	1.47	0.98
4-Methyl-1-octylpyridinium chloride	2.97	2.17	2.28
(ethoxymethyl)ethyldimethylammonium chloride	3.59	3.86	3.61
4-(dimethylamino)-1-hexylpyridinium chloride	0.93	1.19	0.92
benzyldodecyldimethylammonium chloride	0.28	0.51	0.3
3-methyl-1-nonylimidazolium hexafluorophosphate	1.85	1.78	1.88
1-(3-Methoxypropyl)-3-methylimidazolium bis(trifluoromethylsulfonyl)amide	3.34	3.07	3.17
(ethoxymethyl)ethyldimethylammonium bis(trifluoromethylsulfonyl)amide	3.8	3.43	3.64
1-(2-methoxyethyl)pyridinium bis(trifluoromethylsulfonyl)amide	3.19	3.4	3.49
1-(3-methoxypropyl)-1-methylpyrrolidinium bis(trifluoromethylsulfonyl)amide	3.4	3.29	3.47
4-(2-hydroxyethyl)-4-methylmorpholinium bis(trifluoromethylsulfonyl)amide	3.19	3.2	3.69
4-(dimethylamino)-1-hexylpyridinium bis(trifluoromethylsulfonyl)amide	0.93	0.77	0.4
ethyl(2-methoxyethyl)dimethylammonium bis(trifluoromethylsulfonyl)amide	3.31	3.46	3.64
1-butyl-3-methylimidazolium trifluoromethanesulfonate	3	3.36	3.23
1-hexylquinolinium tetrafluoroborate	1.07	1.13	1.38
1-butyl-3-methylimidazolium tetrafluoroborate	3.11	3.07	3.27
1-butyl-4-methylpyridinium tetrafluoroborate	2.98	3.14	3.13
1-(2-ethoxyethyl)-1-methylpiperidinium bromide	4.31	3.85	4.34
1-(2-ethoxyethyl)pyridinium bromide	4.24	3.58	3.97
1-Ethyl-3-methylimidazolium chloride	3.86	3.98	3.83
1-hexyl-3-methylimidazolium chloride	2.82	2.71	2.8
1-butyl-3-methylimidazolium 1-methanesulfonate	3.51	3.3	3.26
1-Ethyl-3-methylimidazolium methylsulfate	4.2	4.11	3.99
1-decyl-3-methylimidazolium hexafluorophosphate	1.5	1.46	1.45
1-heptyl-3-methylimidazolium hexafluorophosphate	2.3	2.43	2.59
3-methyl-1-octylimidazolium hexafluorophosphate	1.96	2.1	2.26
1-hexyl-3-methylimidazolium bis(trifluoromethylsulfonyl)amide	2.24	2.29	2.36
(cyanomethyl)ethyldimethylammonium bis(trifluoromethylsulfonyl)amide	3.87	3.72	3.85
1-(2-hydroxyethyl)pyridinium bis(trifluoromethylsulfonyl)amide	3.79	3.62	3.54
1-(3-hydroxypropyl)pyridinium bis(trifluoromethylsulfonyl)amide	3.55	3.4	3.35
1-(ethoxymethyl)pyridinium bis(trifluoromethylsulfonyl)amide	3.12	3.35	3.44
ethyl(2-ethoxyethyl)dimethylammonium bis(trifluoromethylsulfonyl)amide	3.28	3.06	3.53
ethyl(3-methoxypropyl)dimethylammonium bis(trifluoromethylsulfonyl)amide	3.54	3.15	3.47
1-butyl-3-methylimidazolium toluene-4-sulfonate	3.29	2.93	2.91
3-methyl-1-nonylimidazolium tetrafluoroborate	1.65	1.52	1.52
1-(3-hydroxypropyl)-1-methylpiperidinium bis(trifluoromethylsulfonyl)amide	3.63	3.58	3.47
1-(cyanomethyl)-3-methylimidazolium bis(trifluoromethylsulfonyl)amide	3.9	3.64	3.57
butylethyldimethylammonium bis(trifluoromethylsulfonyl)amide	3.43	3.07	3.4
ethyl(3-hydroxypropyl)dimethylammonium bis(trifluoromethylsulfonyl)amide	3.83	3.31	3.66
1-butyl-1-methylpyrrolidinium tetrafluoroborate	2.9	3.44	3.2
1-(cyanomethyl)-1-methylpiperidinium chloride	4.58	4.35	4.64
1-Ethylpyridinium chloride	4.22	4.04	3.94
1-(2-hydroxyethyl)-3-methylimidazolium tetrafluoroborate	3.48	3.7	3.62
1-(2-hydroxyethyl)-3-methylimidazolium iodide	4.6	4.18	4.07
1-benzyl-3-methylimidazolium chloride	3.3	3.36	3.26
1-benzyl-3-methylimidazolium hexafluorophosphate	3.3	3.35	3.31

1-butyl-3,4-dimethylpyridinium chloride	3.05	3.18	3.19
1-butyl-3,5-dimethylpyridinium chloride	3.42	3.32	3.35
1-butyl-3-methylimidazolium 4-methylbenzenesulfonate	3.29	2.93	2.91
1-butyl-3-methylpyridinium dicyanidoamide	3.46	3.59	3.17
1-butylpyridinium hexafluorophosphate	3.85	3.47	3.57
1-methyl-3-pentylimidazolium hexafluorophosphate	3.07	3.03	3.13
1-butyl-4-methylpyridinium hexafluorophosphate	3.3	3.39	3.48
1-(2-methoxyethyl)-1-methylpiperidinium bromide	4.6	4.26	4.58
4-(2-methoxyethyl)-4-methylmorpholinium chloride	4.6	4.15	4.63
4-(3-hydroxypropyl)-4-methylmorpholinium chloride	4.6	4.11	4.57
1-butyl-3-methylimidazolium bis(trifluoromethylsulfonyl)imide	2.68	2.92	2.78

Table S2: The list of ionic liquids that form the dataset.

References

[1] K. Roy, S. Kar, and P. Ambure, "On a simple approach for determining applicability domain of qsar models," *Chemometrics and Intelligent Laboratory Systems*, vol. 145, pp. 22–29, 2015.

[2] S. Kar, K. Roy, and J. Leszczynski, "Applicability domain: a step toward confident predictions and decidability for qsar modeling," in *Computational Toxicology*, pp. 141–169, Springer, 2018.
